# Buffalo General Hospital

**Published:** 1857-07

**Authors:** 


*Buffalo General Hospital.*—It is with much pleasure that we announce that the workmen are already engaged upon one wing of this new hospital. It is intended to open it for patients during the coming fall or winter. This new institution will add largely to the already excellent clinical advantages of Buffalo. The edifice is situated on High street, about a quarter of a mile from the college. It is intended to be a model building for its purposes.

